# A Multi-Level Approach as a Powerful Tool to Identify and Characterize Some Italian Autochthonous Common Bean (*Phaseolus vulgaris* L.) Landraces under a Changing Environment

**DOI:** 10.3390/plants11202790

**Published:** 2022-10-21

**Authors:** Martina Falcione, Melissa Simiele, Alessandra Renella, Gabriella Stefania Scippa, Paolo Di Martino, Dalila Trupiano

**Affiliations:** Department of Biosciences and Territory, University of Molise, 86090 Pesche, IS, Italy

**Keywords:** agrobiodiversity, phaseolin, ISSR marker, morphological descriptors, plant diversity, stress response

## Abstract

A prime role in matters of agrobiodiversity is held by landraces, which serve as a repository gene pool able to meet sustainable development goals and to face the ongoing challenges of climate change. However, many landraces are currently endangered due to environmental and socio-economic changes. Thus, effective characterization activities and conservation strategies should be undertaken to prevent their genetic and cultural erosion. In the current study, the morphological, genetic, and biochemical analyses were integrated with stress response-related studies to characterize the diversity of seven Italian autochthonous common bean landraces. The results showed that the morphological descriptors and the neutral molecular markers represent powerful tools to identify and distinguish diversity among landrace populations, but they cannot correlate with the stress tolerance pattern of genetically similar populations. The study also supported the use of proline as a biochemical marker to screen the most salt-sensitive bean landraces. Thus, to fully elucidate the future dynamics of agrobiodiversity and to establish the basis for safeguarding them while promoting their utilization, a multi-level approach should always be included in any local and national program for the characterization/conservation/use of genetic resources. This study should represent the basis for further joint research that effectively contributes to set/achieve Italian priorities towards sustainability in the framework of emerging environmental, societal, and economic challenges.

## 1. Introduction

The safeguarding of agrobiodiversity, as an extension of the concept of biodiversity conservation, refers specifically to the preservation of all varieties/landraces of species of agricultural interest. Besides being an integral part of the local traditional knowledge and cultural heritage [1], landraces usually show the best adaptation to the pedoclimatic conditions of restricted geographical areas [2], and they are generally appreciated for their distinctive taste and high nutritional value [3]. Furthermore, they represent a source for lower-input agricultural systems and genetic crop improvement programs, guaranteeing food diversity for humans and other living beings [4,5]. Thus, their conservation and production is vital to enhance the sustainable development in challenging climatic conditions, optimizing the farming systems, and valorizing the marginal areas of Italy [6,7,8].

In this sense, agrobiodiversity represents a relevant challenge for rural marginal area (39% of the agricultural land in Italy) development [9], at both national and regional scales, and in particular, to promote the profitability of mountain agricultural activities—sustainable productions, as well as the diversification of products and services offered by farms—and, thus, the permanence of specific population segments in inland, peripheral rural, and mountainous districts. However, during the last decades, due to complex social, economic, and cultural changes [10] in Italy, as well as in other developed European countries, important locally-adapted sources have been subjected to genetic erosion, reaching a rate of 72.8% in the south of the Italian Peninsula [11]. This phenomenon has prompted governments to take immediate action, assigning economic incentives and subsidies to candidate rural districts (National Strategy for Inner Areas), adhering to international strategies such as the EU Biodiversity Strategy 2020 [12] and the 2030 Agenda for Sustainable Development [13], and drawing up national guidelines for the conservation and enhancement of agrobiodiversity. In this regard, Italy recently recognized the importance of establishing a National Agrobiodiversity Register [14] to collect information on landraces from all Italian regions. However, out of 20 regions, 12 were uncharacterized (having no landraces) [15], although many unknown landraces likely exist, but are isolated on farms. Recently, a more complete list of all Italian herbaceous landraces was produced [16]. Most of these resulted from the *Fabaceae*, *Poaceae*, and *Solanaceae* families and are concentrated in the marginal area (sub-mountain, hilly, and foothill areas; 150–800 m.a.s.l.). The Tuscany region represents the richest region in herbaceous landraces, with 197 varieties, while Molise is the region with the highest density of landraces (number weighted on the area), where there were 50 varieties of common bean (*Phaseolus vulgaris* L.) identified [16]. This richness, also observed in other Apennine areas, is probably determined by a variety of environmental and anthropic factors, such as different bioclimatic levels (e.g., the transition from sea level to mountain altitudes over a short distance), gastronomic heritage, family-based agrosystems, and related low-input practices [17].

In particular, the common bean is an ancient legume crop [18,19], and a typical element of rural economies, allowing for the evolution of many landraces adapted to restricted areas, especially in the southern Italian regions [20]. Although some of common bean landraces are collected and stored in regional seed banks and/or in universities/institutes and facilities for ex situ conservation [21,22], others continue to survive only in marginal areas of several Italian regions through on-farm conservation. Indeed, most of these landraces are severely outdated and endangered due to the advanced age of the farmers who use them, the spreading and wide availability of new commercial varieties, and the socio-cultural context in which they are cultivated. These accessions are often poorly known, and it is therefore of paramount importance to preserve them as part of our heritage (diversity) through a fully effective characterization of each landrace, which would further promote the efforts in planning adequate safeguarding actions.

It is well known that the analysis of seed morphological parameters represents a powerful tool to identify and characterize landraces and discriminate among *P. vulgaris* populations [23,24]. However, in the postgenomic era, molecular markers have emerged as powerful tools for the analysis of germplasm diversity, showing a high rate of reproducibility and efficiency, with no influence from environmental factors [25]. Numerous kinds of genetic markers have been employed for the evaluation of common bean genetic variation, such as inter-simple sequence repeats (ISSR), simple sequence repeats (SSR), random amplified polymorphic DNA (RAPD), amplified fragment length polymorphism (AFLP), and single nucleotide polymorphism (SNP) [2,26,27,28,29,30]. ISSR has proven to be one of the best-suited molecular markers for the accurate assessment of genetic relationships among bean genotypes, with a high efficiency regarding genetic diversity quantification [31,32,33].

The analysis of seed storage proteins represents a valuable contribution to the overall germplasm evaluation and assessment of genetic diversity [34]. Unlike other legumes, the storage proteins in the common bean are mainly constituted by phaseolin, which accounts for 50% of the total proteins in mature seeds. Phaseolin consists of a number of polypeptides (Mr 54–44 kDa), which vary according to their eco-geographical origin and related domestication events. This fact supports the hypothesis that there are two major gene pools within the common bean germplasm, the Mesoamerican and Andean, therefore phaseolin can be considered as a biochemical marker [35].

Furthermore, landraces show a spectrum of responses to different stressors, which are a combined result of complex interactions among different morphological, physiological, and biochemical features [36,37,38], and which also contribute to the description of their genetic diversity. Thus, a multidisciplinary characterization approach, combining morphological, genetic, biochemical, and stress response-related studies, has proven to be a more efficient method of exploring landrace diversity and identifying distinctive landrace traits [15,25,39,40]. This approach may provide a better understanding regarding the genetic resources best suited for use in managing the current climatic variability and adapting to progressive climate changes [41,42].

Based on these premises, in the present study, the diversity of some Italian common bean landraces was assessed and explored by using a multi-level approach able to integrate morphological, genetic, and phaseolin pattern characteristics, along with their ability to counteract two types of stress (salt and osmotic stress) that frequently occur in the Mediterranean Basin and continuously increase due to the changes in the climate and anthropogenic activities.

## 2. Results

### 2.1. Seed Morphological Parameters and Genetic Data

**Figure 1:**
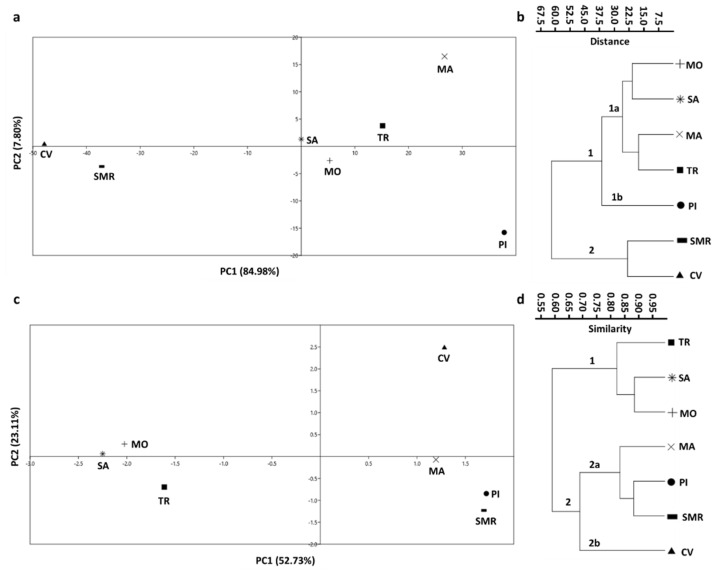
Principal component analysis (PCA) and UPGMA clustering of common bean morphological features and genetic data. Scatter plot of the PCA computed among seven populations of *Phaseolus vulgaris* L., using fifteen morphological features (**a**) and dendrogram resulting from a cluster analysis using the unweighted pair group method with arithmetic mean (UPGMA) with the Euclidean distance index (**b**). Scatter plot of the PCA computed on the seven bean populations, using eight inter-simple sequence repeat (ISSR) primers (**c**) and dendrogram resulting from UPGMA clustering (Jaccard similarity index) (**d**). CV: Ciliegino; SMR: San Michele Rosso; MO: Monachella; MA: Mascherino; PI: Pinto; TR: Tuvagliedda Rossa; SA: Suocera e Nuora.

In our morphological analysis, the main quantitative and qualitative seed morphological descriptors (see Materials and Methods) of seven common bean populations were examined (Table S1; Supplementary Materials). In the principal component analysis (PCA) scatter plot (Figure 1a), Principal Component 1 (PC1) and Principal Component 2 (PC2) accounted for 84.98% and 7.80% of the total variance, respectively. In PC1, two groups could be observed: the first group was comprised of Suocera and Nuora (SA), Monachella (MO), Tuvagliedda Rossa (TR), Mascherino (MA), and Pinto (PI) landraces, while the second group was made up of Ciliegino (CV) and San Michele Rosso (SMR) (Figure 1a). In PC2, MA and PI were well separated from the other populations (Figure 1a). The noted differences were related to seed total area, dark and light-colored areas, weight, and volume.

The dendrogram, resulting from the unweighted pair group method with arithmetic mean (UPGMA) clustering, was in agreement with PCA results and revealed two main clusters (Figure 1b). The first cluster was divided into two subclusters: subcluster 1a contained MO and SA (Euclidean distance ≈ 20), and MA and TR (Euclidean distance ≈ 17). Subcluster 1b was characterized only by PI, which showed ≈ 37.5 Euclidean distance with respect to the other populations of the same cluster. The second cluster contained SMR and CV (Euclidean distance ≈ 25) (Figure 1b).

The genetic relations among the autochthonous common bean landraces were also successively evaluated by using inter-simple sequence repeat (ISSR) markers [33]. The PCA resulted in a scatter plot with PC1 and PC2 scores that accounted for 52.73% and 23.11% of the total variance, respectively. Regarding PC1, two groups were identified: the CV, MA, PI, and SMR populations formed one group, and the TR, SA, and MO (Figure 1c) comprised the other. In PC2, CV was well separated from all the other populations (Figure 1c).

The dendrogram constructed using the UPGMA clustering confirmed the PCA results (Figure 1d). Indeed, two main clusters were identified: TR, SA, and MO were grouped in cluster 1, with the highest genetic similarity between SA and MO (Jaccard similarity index ≈ 0.91); while cluster 2 was divided into two subclusters: MA, PI, and SMR were grouped in subcluster 2a (Jaccard similarity index ≈ 0.81); and CV was assigned to subcluster 2b (Jaccard similarity index ≈ 0.69) (Figure 1d). The ISSR primers generated a number of bands between 4 and 8 (Table S2; Supplementary Materials): LOL2, LOL9, and LOL12 produced the highest number of total bands (NTB: 8), whereas PHV06 and PHV07 exhibited the lowest number (NTB: 4). The PHV06 banding profile was totally characterized by monomorphic bands (NTB: 4 and NMB: 4), with no polymorphism (0.00 % P), while LOL12 (NTB: 8 and NMB: 0) showed the 100.00% P. The values of resolving power (RP) ranged from 0.29 (LOL8) to 5.60 (LOL12), while polymorphism information content (PIC) ranged from 0.03 (LOL8) to 0.44 (LOL12) (Table S3; Supplementary Materials).

### 2.2. Phaseolin Pattern Characterization

A detailed description and interpretation of the phaseolin diversity pattern (Mesoamerican and Andean gene pools) in cultivated common beans of different geographic origins was based on the high-quality 2-DE gel images and the PCR amplification of SCAR phaseolin (Phs), according to the methods of De la Fuente et al. [43].

**Figure 2:**
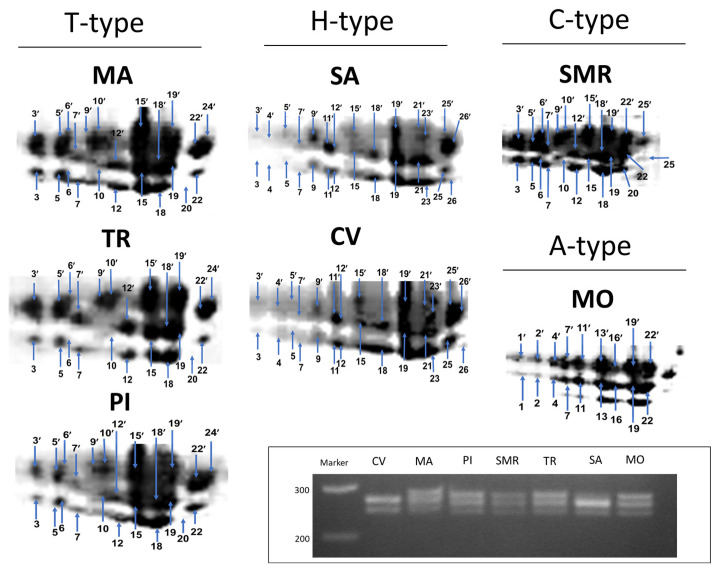
Phaseolin patterns. The 2-DE phaseolin spot patterns of seven common bean populations. Spots were numbered after comparison using the 2-DE maps reported in De la Fuente et al. [44]. Spots of different Mr and similar pI were denoted with and without apostrophes, respectively. The 2-DE imaging was performed using 50 µg of total protein loaded on Bio-Rad 7 cm long IPG strips containing pH 4–7 and 12% polyacrylamide gel. The amplification by PCR of the SCAR marker of the phaseolin in the seven common bean populations is shown in the rectangle. The ladder range is 200–300 base pairs (ApplyChem GmbH, Darmstadt, Germany). CV: Ciliegino; SMR: San Michele Rosso; MO: Monachella; MA: Mascherino; PI: Pinto; TR: Tuvagliedda Rossa; SA: Suocera e Nuora. C: Contender; T: Tendergreen; H: Huevo de Huanchaco; A: Ayacucho.

The 2-DE maps revealed complex phaseolin patterns across the studied landraces (Figure S1; Supplementary Materials), which were characterized by a total of 26 spots that changed within each landrace (Figure 2). In particular, TR, MA, and PI showed a T-type (Tendergreen) phaseolin pattern (spots 3′/3, 5′/5, 6′/6, 7′/7, 9′/9, 10′/10, 12′/12, 15′/15, 18′/18, 19′/19, 22′/22, and 24); SMR and MO showed C-type (Contender) (spots 3′/3, 5′/5, 6′/6, 7′/7, 9′/9, 10′/10, 12′/12, 15′/15, 18′/18, 19′/19, 22′/22, and 25′/25) and A-type (Ayacucho) (spots 1′/1, 2′/2, 4′/4, 7′/7, 11′/11, 13′/13, 16′/16, 19′/19, and 22′/22) phaseolin patterns, respectively; while SA and CV were more similar to H-type (Huevo de Huanchaco) patterns (spots 3′/3, 4′/4, 5′/5, 7′/7, 9′/9, 11′/11, 12′/12, 15′/15, 18′/18, 19′/19, 21′/21, 23′/23, 25′/25 and 26′/26) (Figure 2).

Overall, our 2-DE phaseolin profiles differed from those reported by De la Fuente et al. [43]. Indeed, the new spots that were identified for each population can be attributed to a variety of factors, including improvements in the protein extraction and resolution procedure. Additionally, CV showed some similarities with the Mesoamerican phaseolin pattern founded in the Sanilac (S-type) or Boyacá (B-type) accession used by De la Fuente et al. [43] (Figure 2). These results are fairly in agreement with those obtained by PCR amplification of the Phs SCAR that rendered two major profiles across landraces: two fragments of 249 and 275 bp were noted for CV and SA, typical of S-, B-, or H-type profiles, while three fragments of 249, 275, and 290 bp were obtained for MA, MO, SMR, TR, and PI, characteristic of T-, C-, or A-type profiles (Figure 2).

### 2.3. Morpho-Physiological Characteristics under Stress Conditions

In order to evaluate the effect of stresses on the different common bean landraces, the main plant morphological features, biomass distribution, and relative water content (RWC) were measured after 8 days of plant growth under control, osmotic-, and salt-stressed conditions.

**Figure 3:**
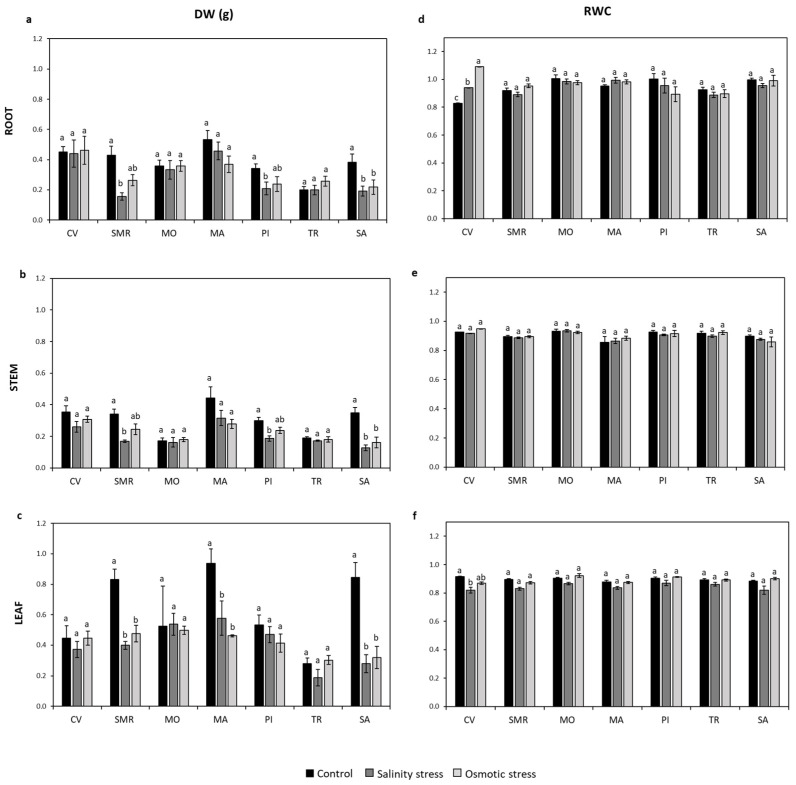
Dry biomass and relative water content. The dry biomass (DW; panel **a**–**c**) and relative water content (RWC; panel **d**–**f**) of roots, stems, and leaves of seven common bean populations, grown under controlled salt and osmotic stress conditions, were measured. Data represent the mean (*n* = 4) ± standard error. A Student’s *t*-test was conducted to weigh the effects of different growth conditions (*p* ≤ 0.05). Mean values marked with the same letter are not statistically different. CV: Ciliegino; SMR: San Michele Rosso; MO: Monachella; MA: Mascherino; PI: Pinto; TR: Tuvagliedda Rossa; SA: Suocera e Nuora.

The analysis of biomass allocation showed that the root (DW_root_) and stem (DW_stem_) dry biomass were decreased in SMR (64% DW_root_ and 51% DW_stem_), PI (40% DW_root_ and 38% DW_stem_), and SA (50% DW_root_ and 63% DW_stem_) by the salt-stress treatment, and only in SA (43% DW_root_ and 67% DW_stem_) by the osmotic-stress condition, when compared to the control plants (Figure 3a,b). In regards to the leaf dry biomass (DW_leaf_), a decline under both types of stress was reported in SMR, MA, and SA (Figure 3c). In detail, in SMR, MA, and SA plants, DW_leaf_ was reduced by 52%, 38%, and 67% under salt stress, and by 43%, 51%, and 62% under osmotic stress, respectively (Figure 3c).

Relative water content (RWC), measured in the three plant organs (roots, stems, and leaves), was proven to be differently affected by salt and osmotic stress. Indeed, the RWC_root_ in CV was increased by 13% and 32% under salt and osmotic stress, respectively. The RWC_stem_ results showed no change compared to the control, and in CV only, a reduction in the RWC_leaf_ (by 11%) was observed under salt stress (Figure 3d–f).

**Figure 4:**
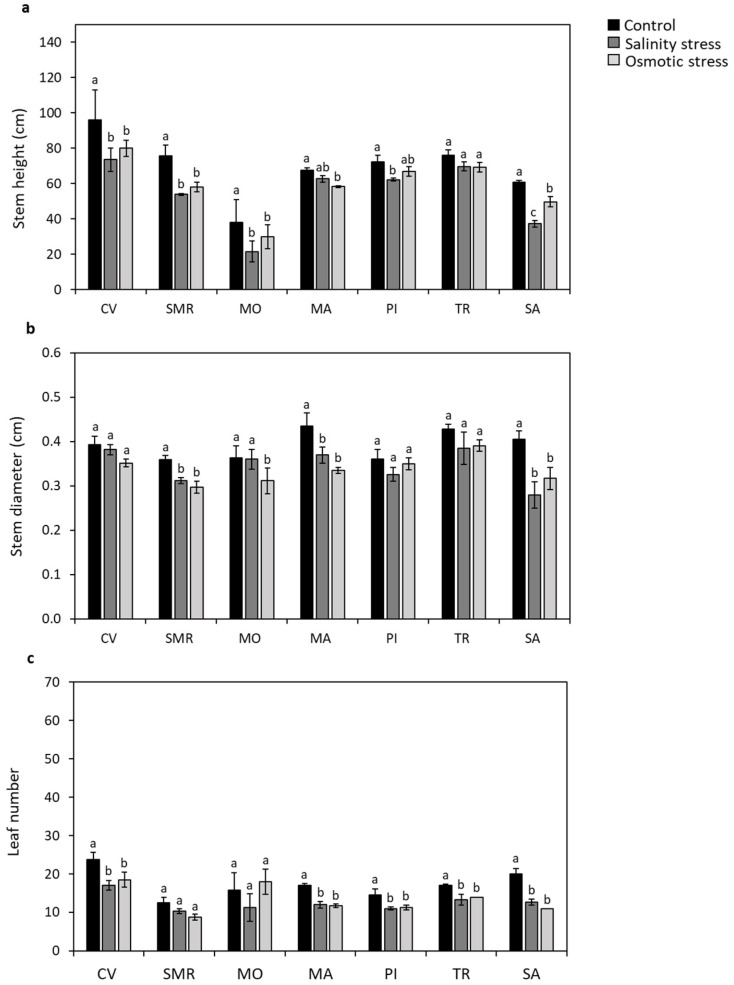
Plant morphological features. Stem height (**a**), diameter (**b**), and leaf number (**c**) of seven common bean populations grown under controlled, salt-, and osmotic-stress conditions. Data represent the mean (*n* = 4) ± standard error. A Student’s *t*-test was conducted to weigh the effects of different treatments (*p* ≤ 0.05). Mean values marked with the same letter are not statistically different. CV: Ciliegino; SMR: San Michele Rosso; MO: Monachella; MA: Mascherino; PI: Pinto; TR: Tuvagliedda Rossa; SA: Suocera e Nuora.

The analysis of the main morphological parameters showed that the stem height decreased by 23% in CV, 43% in MO, 29% in SMR, 14% in PI, and 38% in SA under the salinity stress condition with respect to the control; these reductions were observed under the osmotic stress in CV (20%), MO (22%), SMR (23%), MA (14%), and SA (18%) (Figure 4a). A decline in stem diameter was also reported in SMR, MA, and SA under the salt-stress conditions (with a reduction of 13%, 15%, and 31%, respectively) and osmotic-stress conditions (with a reduction of 17%, 23%, and 22%, respectively). The stem diameter also decreased in MO (14%) under osmotic-stress conditions (Figure 4b). The number of leaves was reduced in CV, MA, PI, TR, and SA under salt-stress conditions (decline of 28%, 29%, 24%, 22%, and 37%, respectively) and under osmotic stress (decline of 22%, 31%, 22%, 18%, and 45%, respectively) (Figure 4c).

### 2.4. Biochemical Analysis

#### 2.4.1. Proline and Malondialdehyde (MDA) Content

**Figure 5:**
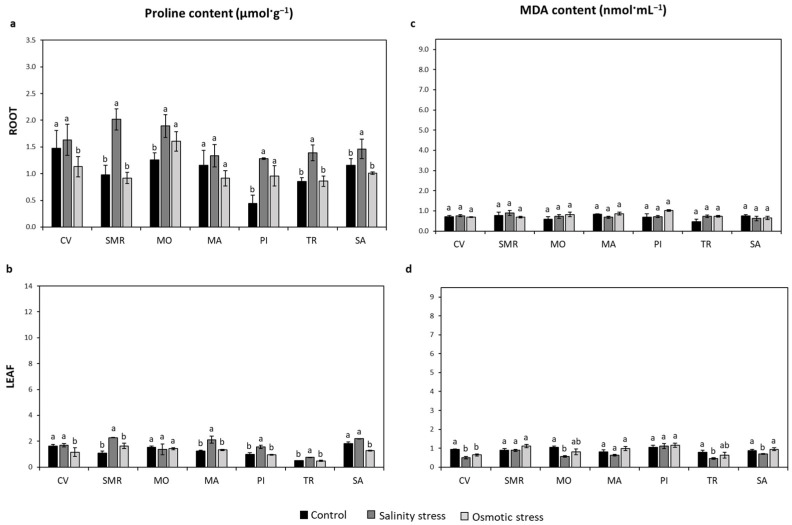
Proline and MDA content. The proline (µmol·g^−1^; panel **a** and **b**) and malondialdehyde (MDA, nmol·mL^−1^; panel **c** and **d**) contents were measured in the root and leaf of seven common bean populations grown under controlled, salt-, and osmotic-stress conditions. Data represent the mean (*n* = 4) ± standard error. A Student’s *t*-test was conducted to weigh the effects of different treatments (*p* ≤ 0.05). Mean values marked with the same letter are not statistically different. CV: Ciliegino; SMR: San Michele Rosso; MO: Monachella; MA: Mascherino; PI: Pinto; TR: Tuvagliedda Rossa; SA: Suocera e Nuora.

Proline was differently distributed in the above- and below-ground parts of common bean populations growing in controlled, salt-, and osmotic-stress conditions. In particular, the salt stress induced an increase in root proline content in SMR (105%), PI (193%), MO (50%), TR (62%), and SA (26%) (Figure 5a). The osmotic stress led to a 23% reduction in CV, while an increase was observed in MO (27%) and PI (116%), compared to the control condition (Figure 5a). The proline was also accumulated in leaves under the salt-stress condition. In detail, proline increased by 108% in SMR and by 68%, 57%, and 52% in MA, PI, and TR, respectively, over the control (Figure 5b). In contrast, the osmotic treatment decreased the leaf proline amount in CV and SA by 29% and 30%, respectively (Figure 5b).

The analysis of MDA content, directly related to oxidative damage, reported no lipid peroxidation in the roots of any of the bean populations subjected to both stress conditions (Figure 5c). Conversely, at the leaf level, compared to the control, the MDA was reduced by 46%, 46%, 42%, and 22%, in CV, MO, TR, and SA, respectively, under salt stress, and by 31% in CV under osmotic stress (Figure 5d).

#### 2.4.2. Photosynthetic Pigment Content

The analysis of the photosynthetic pigments revealed different accumulations of chlorophylls (total Chl A and Chl B) and carotenoids, depending on the bean population considered and the type of stress (Table 1). In detail, the salt stress reduced the total chlorophyll by 27% in CV, while increasing it by 25%, 16%, and 23% in MO, PI, and TR, respectively, compared to the control (Table 1). The Chl A/Chl B was reduced in SMR (8%) and increased in MO (13%), whereas the carotenoids increased by 45%, 23%, 27%, and 27%, in MO, PI, TR, and SA, respectively (Table 1). Under the osmotic-stress condition, the total chlorophyll content was affected only in CV and MO. In detail, it was 17% lower in CV and 20% higher in MO, with respect to the control (Table 1). Furthermore, reduced Chl A/Chl B values were observed in SMR (10%) and PI (14%), while increased Chl A/Chl B values were observed in MO (8%). Finally, the carotenoid content increased by 28% and 23% in MO and SA, respectively (Table 1).

## 3. Discussion

Over the last decades, agrobiodiversity has been jeopardized by several anthropologic pressures, including environmental degradation, rapid changes in land use, and the modernization of agriculture systems based on monoculture farming [44]. This has led to the abandonment of many traditional landraces, inducing a progressive loss of the genetic variability encompassed by the locally adapted germplasms [45], as well as the disappearance of the important traditional knowledge associated with their cultivation [15].

Appropriate characterization activities represent the main tools to assess plant diversity, which, in turn, could help to enhance conservation strategies and to ensure the sustainable use of these valuable plant genetic resources [46]. Furthermore, these activities may provide information about the genetic resources that are best suited for managing the current climatic variability and adapting to progressive climate change [42]. Indeed, landraces show a spectrum of responses to different stressors that are the combined result of the complex interactions among different morphological, physiological, and biochemical features [36,37,38], and which also contribute to the description of their diversity. A wide variety of methods have been used to investigate similarities and relationships among *Phaseolus vulgaris* landraces, and relevant differences were found in Italian common bean populations [47]. However, few studies have evaluated landrace diversity as related to environmental stresses.

Accordingly, a multi-level characterization approach—combining morphological, genetic, biochemical, and stress response-related studies—was used in this study to explore the diversity of the common bean landrace and to identify distinctive climate-smart traits [15,39]. In detail, an integrated approach using morphological, genetic, biochemical, and stress response analyses was used to characterize seven Italian *P. vulgaris* autochthonous landraces in order to (i) investigate the diversity and the relationships among the populations, and (ii) evaluate the stress response under salt- and osmotic-stress conditions, two of the main abiotic stresses occurring in the Mediterranean area.

Phenotypic characterization showed an appreciable morphological variation in seed descriptors (seed size, roundness, shape, color, and type of pattern) that separated the landrace populations into two main groups: one formed by CV and SMR, showing full red coat color, and the other composed of MO, SA, MA, and TR (with MO more similar to SA, and MA more similar to TR), with a bicolored (light and dark) seed coat pattern. The differences were mainly associated with seed coat color patterns and some morphometric descriptors related to seed size and shape.

Although the bicolor seed landrace is more widespread among the Apennine Italian regions, each population is named differently according to the place of cultivation or the morphological features of the seeds, resulting in slight differences. In detail, the SA, PI, and MO populations have all been cultivated by local communities in neighboring geographical areas of Alto Molise (SA: Sant’Angelo del Pesco—805 m.a.s.l.; PI: Agnone—830 m.a.s.l.; MO: Vastogirardi—1200 m.a.s.l.) for at least 50 years. The SA seeds are oval and characterized by white and purple coats, while MO and PI are white and burgundy, with a round and oval shape, respectively. The TR and MA populations, also characterized by seeds with bicolor coats, are cultivated in the other two Italian Apennine Regions. More specifically, TR is a white and mottled red bean landrace cultivated in the hilly areas of Basilicata (Sarconi—636 m.a.s.l.), and it is particularly appreciated for its taste and short cooking time; it is associated with the PGI (Protected Geographical Indication) quality marker. MA is a white and burgundy bean seed cultivated since the late 1800s in a hilly area of Tuscany (Piazza al Serchio—536 m.a.s.l.). In all bicolor landraces, the dark color was predominant, except for in PI, where a larger white area was found (see Table S1; Supplementary Materials).

The CV and SMR landrace populations took their name from the red color of their seed coat. In detail, the CV population is characterized by small, oval, red cherry-colored seeds, and it is typically cultivated only in Vastogirardi Village in the Molise region. SMR is a landrace cultivated in the hilly areas of Basilicata (Sarconi—636 m.a.s.l.), characterized by small, oval, ruby red beans, and which is also associated with the PGI quality marker. Furthermore, only CV exhibits small or medium-size seeds (about 32 g/100-seed weight), while all the other populations are characterized by a larger size (≥40 g/100-seed weight) (see Table S1; Supplementary Materials).

Analyzing the genetic pool of origin, the results confirm that all bean landrace populations derive from the Andean gene pool, as they exhibited the four typical Andean phaseolin characteristics (C-, H-, T-, and A-types). In detail, TR, MA, and PI showed the T-type phaseolin pattern, SA and CV exhibited the H-type pattern, SMR expressed the C-type pattern, and MO presented the A-type pattern. The PCR-based SCAR marker of phaseolin confirmed that all of the landraces belonged to the Andean genetic pool, revealing two bands typical of the H-type in the case of CV and SA and three bands for SMR, TR, MO, MA, and PI, common in the C-, T-, and A-type gene pools. This result supports the proven predominance of the Andean genotypes over the Mesoamerican examples in the Mediterranean Basin. Furthermore, a prevalence of C-type over T-type phaseolin has been reported in Italian and European common bean landraces [48,49], while the H-type phaseolin has proven to be more unusual among the common bean populations in Europe [22]. Moreover, it has been found that cultivars belonging to the Mesoamerican gene pool show small or medium-sized seeds (<25 g or 25–40 g/100-seed weight), while their Andean counterparts have larger seeds (>40 g/100-seed weight) [22]. This evidence is not completely supported by our data, in which the 100-seed weight of CV, SMR, and SA, associated with the Andean gene pool, was 32.2 g, 39.9 g, and 40.0 g, respectively (see Table S1; Supplementary Materials); however, similar exceptions were also found by Logozzo et al. [48] and Piegiovanni et al. [50].

Genetic data, obtained by using ISSR molecular markers, differentiate the previously morphological and phaseolin related landraces into two new main groups. Collectively, TR, SA, and MO were grouped together, with a higher genetic similarity observed between SA and MO, while MA, PI, and SMR were grouped together, and CV stood alone. Although ISSR molecular markers can give us a sense of the patterns and the extent of diversity found among landrace populations [10,51], the small amount of polymorphic loci data assayed in this study might only provide a rough indication of genetic relationships among landraces, which could be further analyzed on a smaller scale. 

The frequent practice of seed exchange among the farmers [50], especially in central Italy, where the exchange of seed materials occurred frequently over the years, following transhumance pathways [51], could explain the higher genetic similarity within the *P. vulgaris* landraces originating from different Italian regions. This practice generally affects the genetic structure of the germplasm, resulting in a closer relatedness with populations cultivated in different Italian regions. In this regard, the Molise region, as a geographically central transhumance region, can act as a connecting element of the Italian Apennine regions [52].

Analyzing the bean landrace populations’ ability to tolerate salt and osmotic stress, landraces displayed specific morpho-physiological and biochemical changes in the three plant organs (leaf, stem, and root), which were strictly related to the genotype and type of stress (salt or osmotic stress). In detail, salt stress adversely affected the overall growth of the SMR and SA landrace populations, reducing biomass accumulation in all three organs. Furthermore, it negatively impacted the root and stem biomass accumulation in PI, but only the leaf biomass in MA populations. The negative effect of salt stress on biomass accumulation was found to be strictly correlated with the high levels of proline in the different organs of the affected populations. On the contrary, osmotic stress only impacted the overall biomass accumulation of the SA landrace population and the leaf biomass of SMR and MA. No correlation with proline content was found. Depending on the landrace, a decrease in stem and leaf biomass was also associated with a decrease in stem diameter, branching, and/or height, as well with a decrease in leaf number and/or area. It is widely reported that salt stress has a higher impact on plant growth and development compared to osmotic stress [53], since it is responsible for both osmotic (cell dehydration) and toxic (ions accumulation) effects on plant cells, impairing several morpho-physiological parameters of plants [54]. Several studies reported a positive correlation between proline accumulation and stress tolerance in plants [55,56]. However, in some plant species, and in particular, in *P. vulgaris* plants, proline levels were higher in salt stress-sensitive cultivars compared to salt stress-tolerant examples [57,58]. These authors assumed that proline accumulation is a symptom of injury, rather than an indicator of salinity resistance, and that its biosynthesis presumably occurred as a consequence of disturbance in cell homeostasis, reflecting damage in response to salt stress.

However, the negative impact of salt or osmotic stress on plant growth was not associated with oxidative stress, as reported by the unchanged or even decreased MDA contents in the leaves and root of the populations with impaired growth.

It is reported that the accumulation of proline can alleviate membrane damage and reduce MDA levels in plants subjected to stress conditions [59]. However, proline is not only functional in preventing lipid membrane peroxidation, but it is also involved in other physiological functions when plants experience stress. As these functions include osmoprotection, free radical scavenger activity, macromolecule protection from denaturation, regulation of cytosolic acidity, and regulation of programmed cell death, proline homeostasis is essential for generating energy for metabolically demanding cells [60]. It is therefore likely that its importance is related to its ability to integrate growth/development according to environmental cues, and that the plant must develop specific strategies to effectively channel proline for optimizing the growth–defense tradeoffs. However, while there was an unequivocal association of higher proline contents with growth inhibition induced by salt stress, it was uncorrelated with osmotic stress. Indeed, in the SA landrace population, despite osmotic stress inducing a reduction in plant growth parameters (biomass and morphological traits), the proline content remained unchanged. However, plant stress responses may vary, considering the type, intensity, and duration of the stress. Moreover, even though stress tolerance mechanisms are based on specific stress responses, not all responses are relevant for tolerance. Proline clearly plays more than one role in the plant, and other aspects are important in the regulation of proline metabolism. For example, a partial catabolization of proline to pyrroline-5-carboxylate (P5C), which is toxic for certain tissues and leads to apoptosis, could be hypothesized to affect the overall plant growth [61].

Thus, proline accumulation could be considered as a general response to salt stress, rather than a tolerance mechanism. The unchanged proline content observed in the CV, MO, and TR populations, which are tolerant to both salt and osmotic stress, confirmed this hypothesis. Thus, other ion osmotic adjustments, including those related to K^+^, Na^+^, Cl^−^, and organic osmolyte (sugar alcohols or ammonium compound), could also fulfill osmoprotective functions [62].

Furthermore, the involvement of an effective antioxidant defense system, composed of enzymatic and non-enzymatic components, could combat the harmful effects of oxidative stress on the cellular components [63]. Among different redox-balancing agents, it could be assumed that carotenoids, along with chlorophyll, could act as defense molecules against stress [64]. This evidence corresponds with the higher levels of chlorophylls and carotenoids found in MO and TR landraces. Indeed, carotenoids, besides representing essential pigments in photosynthesis, also play a major role in oxidative stress tolerance, since they protect the photosynthetic apparatus by quenching harmful free radicals, formed naturally during photosynthesis and under exposure to stress conditions [65]. Total chlorophyll content and/or Chl A/Chl B ratios were also altered under stress conditions, as a means of maximizing photosynthetic efficiency [66].

The CV bean populationwas not affected by salt or osmotic adversity in term of biomass accumulation but showed a decrease in RWC and proline amounts at root and leaf level, as well as a decrease in total chlorophyll. The RWC is an important indicator of water status in plants because it reflects the balance between water supply to the tissues and transpiration rate [67]. Reductions in RWC under salt and osmotic stress, caused by low osmotic potential, are commonly reported in the literature [68,69]. In detail, it has been argued that salt-tolerant plants decrease the hydraulic conductance of their roots, thereby reducing the delivery of (salty) water to the shoot and resulting in reduced water potential in their leaves [70]. The osmotic-induced low water potential could also accelerate the degradation of structural proteins [71], from which most of the osmolytes are synthesized. This could explain why a decrease in both RWC and proline amounts was observed in CV leaves, and a negative correlation was observed in CV roots (high RWC and low proline). However, other active (augmentation of other solutes within the cells, such as sugar or ammonium compounds) and/or passive (loss of cell water) osmotic adjustments could play a role in stress response—allowing plant tissues to retain water, even at low water potentials, maintaining turgor—and indirectly, in growth and productivity under water deficit conditions [72].

The lower amounts of chlorophyll could be interpreted as a tolerant trait and, in particular, an alternative route to produce H_2_O_2_ by the photocatalytic activity of chlorophyll itself, which acts as a secondary messenger in various stress-responsive signaling pathways, and which have been found essential for “salt stress preparedness” in tolerant plant species [73,74].

## 4. Materials and Methods

### 4.1. Phaseolus vulgaris Landraces

The seeds of seven common bean (*Phaseolus vulgaris* L.) landraces, cultivated in the Molise region (Italy), and in other two Italian Apennine regions (Tuscany and Basilicata) were analyzed (Figure S2; Supplementary Materials). Specifically, four landraces were collected from local growers in the geographical areas of Alto Molise, represented by the Vastogirardi (Ciliegino, CV, and Monachella, MO), Agnone (Pinto, PI), and Sant’Angelo del Pesco (Suocera e Nuora, SA) municipalities of the rural districts of the Alto Medio Sannio Inner Area [75]. All these municipalities are considered by the National Strategy for Inner Areas (SNAI), which aims to fight the depopulation and further marginalization of Italian inland areas by promoting territorial development and cohesion. One landrace, called Mascherino (MA), was collected from a single farm located in Piazza al Serchio (LU, Tuscany region, Italy) and, the two others, Tuvagliedda Rossa (TR) and San Michele Rosso (SMR), awarded in Italy with the PGI (Protected Geographical Indication) quality marker, were purchased from Belisario Farm (Sarconi, PZ, Basilicata region, Italy). All the seeds, collected from different landrace populations, were divided into two lots: one assigned to the Germplasm Bank of the University of Molise for long-term storage, and the other used for seed multiplication and for future reference.

### 4.2. Seed Morphological Traits

A total of fifteen morphological parameters were measured for each common bean seed population. In detail, quantitative morphological parameters, such as area (mm^2^), perimeter (mm), major axis length (mm), minor axis length (mm), roundness, dark-colored seed area (mm^2^), and light-colored seed area (mm^2^), were measured from digital images using the software Image J (Version 1.51i Wayne Rasband-NIH; https://rsb.info.nih.gov/ij/ accessed on 20 January 2022), while 100-seed weight (g), 100-seed volume (mL), and density (g·mL^−1^) were measured with a precision balance and a graduated cylinder.

Qualitative seed descriptors were visually assessed using Image J software analyzing the following characteristics: seed coat pattern (absent, bicolor, spotted bicolor), number of seed coat colors (one, two, more than two), primary/main seed coat color (red, white, dark), predominant secondary seed coat color (none, white, dark), and seed shape (round, oval) [76,77].

Principal component analysis (PCA) was performed to define the role of each morphological characteristic in the grouping of accessions. Cluster analysis was also conducted by UPGMA (unweighted pair group methods using arithmetic averages) analysis to create a dendrogram with Past Version 4.03 software.

### 4.3. DNA Analysis

For each landrace population, seeds were germinated in a growth chamber under controlled conditions (25 °C), and total genomic DNA was extracted from five individuals (youngest leaf of 10-day-old seedlings); the samples were processed separately. The DNA extraction was performed using the Invisorb^®^ Spin Plant Mini Kit (Stratec Molecular GmbH, Berlin, Germany). DNA was quantified by measuring the absorbance at 260 nm on a spectrophotometer, and individual stock concentrations were adjusted to 20 ng·µL^−1^ for PCR. A total of 8 inter-simple sequence repeat (ISSR) primers, reported in Marotti et al. [2] and Peña-Ortega et al. [28], were used for the genetic analysis (Table S3; Supplementary Materials). Amplification reactions were carried out in volumes of 25 µL, containing H_2_O MilliQ, 20 ng·µL^−1^ template DNA, 1 unit of GOTaq DNA polymerase (Promega, Madison, WI, USA), 2.5 mM each of dNTP (Sigma-Aldrich, St. Louis, MO, USA), 25 mM MgCl2 (Promega, Madison, WI, USA), and 10 µM primer (Invitrogen, Thermo Fisher Scientific, Waltham, MA, USA) in 5x Green color Go Taq Flexi buffer (Promega).

Polymerase chain reactions (PCRs) were run in a T100 Thermal Cycler (Bio-Rad, Hercules, CA, USA) under the following conditions: 3 min at 94 °C, for initial denaturation, 36 cycles of 45 s at 94 °C for denaturation, 1 min at annealing temperature, 1 min at 72 °C for extension, followed by 5 min at 72 °C for a final extension of the single strands. ISSR-amplified fragments were resolved on a 1.5% agarose gel stained with ethidium bromide and visualized under UV light. Gels were scanned with ChemiDoc (Bio-Rad), and amplification profiles were analyzed with the Quantity-One Band Analysis software (Bio-Rad). Bands were scored according to presence or absence, and the raw data were processed with Past Version 4.03 software to obtain a standardized matrix and perform PCA and UPGMA clustering (Jaccard’s similarity index). Finally, for each ISSR marker, the number of total bands (NTB), number of monomorphic bands (NMB), number of polymorphic bands (NPB), percentage of polymorphic bands (% P), resolving power (RP), and polymorphism information content (PIC) were calculated, according to the methods of Abdelaziz et al. [78].

### 4.4. Phaseolin Analysis

#### 4.4.1. Protein Extraction and Two-Dimensional Electrophoresis (2-DE) Separation

The total proteins were extracted from three independent samples (1.0 g of a pool of 30 dry seeds), as described by Scippa et al. [79]. The protein quantity in the extracts was measured following the Bradford method, using bovine serum albumin as the standard.

For isoelectric focusing (IEF) analysis, immobilized pH gradient (IPG) strips (7 cm pH 4–7, Bio-Rad) were rehydrated overnight with 125 mL of rehydration buffer [6 M urea, 2% (*w*/*v*) CHAPS, 0.5% (*v*/*v*) Triton X-100, 20 mM dithiothreitol (DTT) and 1% (*w*/*v*) carrier ampholytes pH 3–10] containing 50 µg of total proteins to obtain the best resolution of the area of the 2-DE gels, wherein the phaseolin polypeptides are located [43].

Isoelectric focusing (IEF) was performed with the PROTEAN IEF Cell system (Bio-Rad) at 12 °C at the following voltages: 250 V (90 min), 500 V (90 min), 1000 V (180 min), and 8000 V, for a total of 55 kVh. Focused strips were incubated in the equilibration solution (50 mM Tris pH = 8.8, 6 M urea, 30% glycerol, and 2% SDS) with 2% dithiothreitol (DTT) for 20 min at room temperature and then with 2.5% iodoacetamide (IAA) for the same time and under the same conditions.

Two-dimensional electrophoresis was carried out using a Protean apparatus (Bio-Rad) and 12% polyacrylamide gels (30% Acrylamide mix, 1.5M Tris pH 8.8, 10% SDS, 10% Ammonium persulfate, Temed), with 120 V applied for 2–3 h. Each sample was run in triplicate. Standard proteins (Bio-Rad) were used to estimate the molecular weight of the protein spots.

Gels were fixed for 1 h with a solution of ethanol 40% and acetic acid 10%, stained for 16–18 h with Brilliant blue G-Colloidal Concentrate (Sigma Aldrich), rinsed for 4 days, and scanned using a Chemi Doc (Bio-Rad) device. Image analysis was performed using the PDǪuest software, version 8.8 (Bio-Rad). Spot detection and matching between gels were performed automatically, followed by manual verification. After normalization of the spot densities against the whole-gel densities, the percentage volume of each spot was averaged for the three different replicates for each gel.

#### 4.4.2. Amplification of the Phaseolin (SCAR) Marker by PCR

Amplification of the sequence-characterized amplified region (SCAR) marker of the phaseolin seed protein (Phs) locus was carried out by the PCR on three individuals in each population. Amplification reactions were carried out in volumes of 25 µL, containing H_2_O MilliQ, 20 ng·µL^−1^ template DNA, 2 units of GOTaq DNA polymerase (Promega), 2.5 mM of dNTP (Sigma-Aldrich), 25 mM MgCl_2_ (Promega), and 10 µM primer (Invitrogen, Thermo Fisher Scientific) in 5x Green color Go Taq Flexi buffer (Promega, WI, USA).

The sequence for the upstream Phs primer is 5′-AGCATATTCTAGAGGCCTCC-3′, and the sequence for the downstream Phs primer is 5′-GCTCAGTTCCTCAATCTGTTC-3′. The PCR primers were selected from regions of complete identity between the T and S phaseolin sequences [80] covering the region where the 15 bp repeat (present in the a-type genes) and a 21 bp direct repeat (third intron) are located.

PCR reactions were run in a T100 Thermal Cycler (Bio-Rad) under the following conditions: 3 m at 94 °C, for initial denaturation, 40 cycles of 94 °C for 30 s, 55 °C for 30 s, and 72 °C for 30 s, followed by 5 min at 72 °C for a final extension of the single strands. Amplified fragments were resolved on a 2.5% agarose gel along with a 100–1000-bp ladder (ApplyChem GmbH, Darmstadt, Germany), stained with ethidium bromide, and visualized under UV light. The gels were scanned with a ChemiDoc (Bio-Rad) device.

### 4.5. Plant Growth Conditions and Stress Treatments

Seeds of each population were germinated in plateau pots on moistened vermiculite and placed in a growth chamber under controlled conditions (16 h light/8 h dark cycle under 25 °C temperature) for 20 days.

Four plants for each population were successively transplanted on a moistened mixture of soil and vermiculite (2:1) in 0.5 L pots (diameter = 11 cm) placed in plastic trays (12 pots per tray), and grown under different conditions: (i) control—plants were irrigated with 50 mL of distilled water; (ii) salt stress—plants were irrigated with 50 mL of 200 mM NaCl solution; and (iii) osmotic stress–plants were irrigated with 50 mL of 180 mM mannitol solution. The salt and osmotic concentrations were chosen to reproduce the osmotic potential of 0.8 MPa for both conditions [81]. The solutions were renewed every day for 8 days, and all plants were grown in an environmental chamber under the following controlled conditions: long-day photoperiod (16 h of light and 8 h of darkness), light intensity yield of approximately 250–300 µmol·m^−2^·s^−1^ (light meter sensor—HD2302.0—Delta Ohm; Caselle di Selvazzano, Italy) at pot height, a temperature of 23 °C, and a relative humidity range between 40–50%. Plant material (roots, stems, and leaves) was harvested after 8 days of growth and subjected to further analysis.

### 4.6. Plant Morpho-Physiological Analysis

Plant morphological analyses were performed by measuring the stem height and the leaf number, and by using ImageJ Version 1.51i software (Wayne Rasband-NIH https://rsb.info.nih.gov/ij/ accessed on 20 January 2022) to measure the stem diameter. 

In addition, leaf, stem, and root biomass allocations were determined after two days of drying in an oven at 80 °C (dry weight, DW), and the relative water content (RWC) of the three organs (root, stem, and leaf) was also calculated using the formula reported in Smart and Bingham [82]: [(FW—DW)/(TW—DW)]·100.

All the measurements were performed on four plants and expressed as mean ± standard error. The statistical differences among treatments and populations were determined through a Student’s *t*-test, and significance was accepted at *p* ≤ 0.05.

### 4.7. Biochemical Analysis

#### 4.7.1. Proline Content

Proline content was measured in the roots and leaves according to the method of Carillo and Gibon [83], with some modifications. Briefly, plant material (0.05 g), powdered with liquid nitrogen, was homogenized in 1 mL of 70% ethanol, incubated at 95 °C for 20 min, and cooled in an iced bath. The mixture was centrifuged (10 min, 14,000× *g*), and the supernatant was recovered and stored at −20 °C. 

For proline determination, 500 μL of extract (supernatant) was added to 500 μL of reaction mix (50% of 2% ninhydrin and 50% of 60% glacial acetic acid); the solution was incubated at 95 °C for 20 min, cooled in an iced bath, centrifuged (10 min, 10,000 rpm), and allowed to stand for 24 h in the dark at 4 °C. Next, the samples were centrifuged (15 min, 10,000 rpm), and their absorbance was determined by a spectrophotometer (Bio-Rad) at 520 nm. The proline concentration was determined as mean ± standard error of triplicate measurements using a standard curve and expressed as μmol·g^−1^ using the equation reported by Carillo and Gibon [83].

The statistical differences among treatments and populations were determined through the Student’s *t*-test, and significance was accepted at *p* ≤ 0.05.

#### 4.7.2. Lipid Peroxidation Assay

Lipid peroxidation in the roots and leaves was determined by using the thiobarbituric acid (TBA) reaction, followed by the measurement of malondialdehyde (MDA) content, as reported by Ben Abdallah et al. [84]. Plant material (0.05 g), powdered with liquid nitrogen, was extracted with 0.5 mL of 0.1% trichloroacetic acid (TCA). The samples were centrifuged at 15,000× *g* for 10 min, and 250 μL of supernatant was added to 1 mL of 0.5% TBA prepared in a 20% TCA solution. The solutions were incubated at 95 °C for 30 min, vortexed, cooled in an ice bath, and then centrifuged at 10,000× *g* for 15 min. The specific absorbance of the supernatant was measured at 532 nm, while the non-specific absorbance was measured at 600 nm. The MDA concentration was expressed as nmol·mL^−1^ (mean ± standard error of triplicate measurements) and calculated as reported by Hodges et al. [85].

The statistical differences among treatments and populations were determined through the Student’s *t*-test, and significance was accepted at *p* ≤ 0.05.

#### 4.7.3. Photosynthetic Pigment Content

The total chlorophyll (Chl), the Chl A/Chl B ratio, and the carotenoid contents were determined spectrophotometrically in the leaf tissues, according to the method reported by Polzella et al. [86]. Briefly, fresh leaves (0.1 g), powdered with liquid nitrogen, were homogenized in 1.8 mL of N, *N*-dimethylformamide (DMF), and stored for 48 h at 4 °C. The samples were centrifuged at 14,000× *g* for 5 min and their absorbance was determined at 664 nm, 647 nm, and 480 nm. Photosynthetic pigment content was expressed as μg·mg^−1^ of fresh weight, and as the mean ± standard error of triplicate measurements.

The statistical differences among treatments and populations were determined through the Student’s *t*-test, and significance was accepted at *p* ≤ 0.05.

## 5. Conclusions

The present study demonstrates that a multi-level characterization approach, combining morphological, genetic, biochemical, and stress response-related studies, proves to be an efficient method to explore landrace diversity and identify climate-smart distinctive traits in these dynamic populations.

Indeed, relevant information was obtained on the diversity of some Apennine common bean landraces, as well as their ability to tolerate salt and osmotic stresses. The genetic distance among landrace populations did not correlate with their stress tolerance level. In particular, the CV and MO population, cultivated in the Molise region, and TR, cultivated in Basilicata region, were found to be salt-tolerant, whereas MA (Tuscany region), SMR (Basilicata region), and SA and PI (Molise region) were found to be the most sensitive to these stresses. The osmotic stress negatively impacted only the SA landrace population. The practice of frequent exchange among the farmers of central Italy could have affected the genetic structure of the germplasm and/or the plant plasticity, resulting in a closer relatedness with populations cultivated in different Italian regions. Furthermore, the results suggest that proline could be used as a large-scale biochemical screening marker for salt-sensitive bean landraces; however, it is not suitable for use in the case of osmotic stress. Other biochemical traits, such as the amount of sugar alcohols, ammonium compounds, or redox-balancing agents, could be used as general biomarkers of bean stress tolerance. The screening and identification of other common bean landrace populations susceptible or tolerant to salinity and osmotic stress are of great interest for identifying climate-smart landraces for increasing the productivity of staple food crops in stressful environments, which is of interest for plant adaptation to climate change and for the maintenance of yield stability under marginal conditions.

However, short-term and long-term field experiments should be conducted to monitor whether prolonged exposure to salt and osmotic stress may prevent plant growth and to determine whether plant priming could be a complementary and useful strategy to mitigate the injurious effects of salt and osmotic stress. More in-depth “omics” studies will be needed to shed light on the molecular mechanisms adopted by these locally adapted genetic plant resources to cope with salinity and osmotic stresses and to identify important traits useful for plant tolerance/adaptation to the ongoing climate change.

Further investigation should be also planned to explore the nutritional potential of different common bean landrace populations, characterizing the chemical diversity of their seeds and evaluating the possible presence of specific phytochemical markers of landraces and/or nutraceutical compounds important for human health. Starting from these suggestions, agrobiodiversity valorization could represent an effective tool for encouraging models for the sustainable production and use of food. The results of this study could also promote the cultural and socioeconomic value of the marginalized Apennine Italian areas in which these genetic resources are cultivated, transforming currently marginal areas into productive lands.

## Figures and Tables

**Table 1:** Leaf photosynthetic pigment content. Total chlorophyll (Chl) content (µg·mg^−1^), chlorophyll A and B ratio (Chl A/Chl B), and carotenoid content (µg·mg^−1^) of seven common bean populations grown under control, salt-, and osmotic-stress conditions. Data represent the mean (*n* = 4) ± standard error. A Student’s *t*-test was conducted to weigh the effects of different treatments (*p* ≤ 0.05). Mean values marked with the same letter are not statistically different. CV: Ciliegino; SMR: San Michele Rosso; MO: Monachella; MA: Mascherino; PI: Pinto; TR: Tuvagliedda Rossa; SA: Suocera e Nuora.

		Control	Salinity Stress	Osmotic Stress
Total Chl (µg·mg^−1^)	CV	1.34 ± 0.05 a	0.98 ± 0.04 b	1.11 ± 0.02 b
	SMR	1.12 ± 0.06 a	1.13 ± 0.14 a	1.08 ± 0.03 a
	MO	0.92 ± 0.02 b	1.14 ± 0.06 a	1.09 ± 0.03 a
	MA	1.61 ± 0.09 a	1.53 ± 0.06 a	1.72 ± 0.10 a
	PI	2.19 ± 0.08 b	2.56 ± 0.03 a	1.98 ± 0.17 b
	TR	1.41 ± 0.03 b	1.73 ± 0.03 a	1.15 ± 0.11 b
	SA	1.21 ± 0.06 a	1.41 ± 0.11 a	1.39 ± 0.09 a
Chl A/Chl B	CV	3.28 ± 0.05 a	3.22 ± 0.08 a	3.27 ± 0.01 a
	SMR	4.02 ± 0.05 a	3.71 ± 0.10 b	3.57 ± 0.07 b
	MO	3.19 ± 0.38 b	3.63 ± 0.26 a	3.44 ± 0.04 a
	MA	3.39 ± 0.04 a	3.22 ± 0.05 a	3.34 ± 0.04 a
	PI	3.54 ± 0.03 a	3.62 ± 0.27 a	3.02 ± 0.33 b
	TR	3.54 ± 0.02 a	3.33 ± 0.01 a	3.43 ± 0.04 a
	SA	3.23 ± 0.03 a	3.18 ± 0.03 a	3.28 ± 0.07 a
Carotenoids (µg·mg^−1^)	CV	0.19 ± 0.01 a	0.18 ± 0.01 a	0.19 ± 0.00 a
	SMR	0.16 ± 0.01 a	0.18 ± 0.02 a	0.15 ± 0.01 a
	MO	0.12 ± 0.00 b	0.18 ± 0.01 a	0.16 ± 0.01 a
	MA	0.26 ± 0.02 a	0.25 ± 0.01 a	0.26 ± 0.02 a
	PI	0.31 ± 0.01 b	0.39 ± 0.01 a	0.29 ± 0.03 b
	TR	0.23 ± 0.01 b	0.29 ± 0.00 a	0.18 ± 0.02 b
	SA	0.17 ± 0.01 b	0.21 ± 0.02 a	0.21 ± 0.01 a

## Data Availability

Not applicable.

## Supplementary Materials

The following supporting information can be downloaded at: https://www.mdpi.com/article/10.3390/plants11202790/s1, Table S1: Morphological descriptors and analysis; Table S2: ISSR banding profile in common bean landraces; Table S3: Primers used for ISSR analysis and their respective markers’ performance indexes; Figure S1: The 2-DE profiles of common bean landraces; Figure S2: Autochthonous Italian common bean seed accessions.
