# Supplementary material for: A Multi-Level Approach as a Powerful Tool to Identify and Characterize Some Italian Autochthonous Common Bean (Phaseolus vulgaris L.) Landraces under a Changing Environment
Source: Plants (Basel). 2022 Oct 21;11(20):2790. doi: 10.3390/plants11202790 (PMC9609626; doi:10.3390/plants11202790)
Supplement: Supplementary file 1 [file plants-11-02790-s001.zip › plants-1953990-supplementary.pdf]

Supplementary Materials

Table S1. **Morphological descriptors and analysis.** Quantitative and qualitative seed morphological parameters measured for each common bean population. Data represent the mean (n = 10) ± standard error. CV: Ciliegino; SMR: San Michele Rosso; MO: Monachella; MA: Mascherino; PI: Pinto; TR: Tuvagliedda Rossa; SA: Suocera e Nuora.

Population name	Source	100 seed weight (g)	100 seed volume (mL)	100 seed density (g·mL $^{-1}$)	Total area (mm²)	Perimeter (mm)	Roundness	Major axis lenght (mm)	Minor axis lenght (mm)	Dark coloured seed area (mm²)	Light coloured seed area (mm²)	Number of seed coat colours	Main seed coat colour	Predominant secondary seed coat colour	Seed shape	Seed coat pattern
CV	Vastogirardi (Molise) 41° 46' 9.651" (N) 14° 15' 28.894" (E)	32.17 ± 1.75	51.33 ± 5.93	0.64 ± 0.05	60.40 ± 3.84	30.32 ± 0.90	6.32 ± 0.27	10.37 ± 0.30	7.02 ± 0.29	60.40 ± 3.84	0 ± 0	one	red	none	oval	absent
SMR	Sarconi (Basilicata) 40° 14' 38.04" (N) 15° 51' 55.439" (E)	39.90 ± 2.02	72.00 ± 3.46	0.55 ± 0.00	55.15 ± 2.64	28.15 ± 0.62	7.68 ± 0.21	9.64 ± 0.30	7.40 ± 0.28	55.15 ± 2.64	0 ± 0	one	red	none	oval	absent
MO	Vastogirardi (Molise) 41° 46' 50.074" (N) 14° 17' 44.116" (E)	51.57 ± 3.09	80.00 ± 3.06	0.64 ± 0.02	67.75 ± 1.67	31.47 ± 0.39	7.96 ± 0.31	10.42 ± 0.35	8.44 ± 0.22	48.88 ± 2.38	43.15 ± 3.43	two	dark	white	round	bicolor
MA	Piazza al Serchio (Tuscany) 44° 10' 26.828" (N) 10° 18' 42.901" (E)	60.20 ± 1.99	92.67 ± 1.76	0.65 ± 0.01	94.48 ± 3.37	37.19 ± 0.67	7.29 ± 0.25	12.47 ± 0.24	9.39 ± 0.22	48.11 ± 4.78	46.82 ± 3.04	two	dark	white	oval	bicolor
PI	Agnone (Molise) 41° 51' 1.076" (N) 14° 20' 45.679" (E)	57.90 ± 2.68	92.67 ± 3.53	0.62 ± 0.01	82.36 ± 3.88	34.63 ± 0.82	7.21 ± 0.23	12.38 ± 0.40	9.14 ± 0.31	21.02 ± 3.41	60.91 ± 5.68	two	white	dark	oval	bicolor
TR	Sarconi (Basilicata) 40° 14' 38.04" (N) 15° 51' 55.439" (E)	57.67 ± 0.22	86.67 ± 0.67	0.67 ± 0.01	81.94 ± 2.74	33.94 ± 0.55	7.92 ± 0.19	11.72 ± 0.35	9.56 ± 0.18	42.46 ± 3.42	40.79 ± 3.02	more than two	dark	white	oval	spotted bicolor
SA	Sant'Angelo del Pesco (Molise) 41° 54' 18.619" (N) 14° 16' 49.367" (E)	39.97 ± 1.36	77.33 ± 2.40	0.52 ± 0.01	81.06 ± 4.93	33.91 ± 0.93	7.63 ± 0.23	11.95 ± 0.41	8.98 ± 0.26	42.71 ± 4.13	32.24 ± 2.45	two	dark	white	oval	bicolor

Table S2. ISSR banding profile in common bean landraces. The banding profile of each ISSR primers (LOL2, LOL7, LOL8, LOL9, LOL10, LOL12, PHV06 and PHV07) transformed into binary matrix (1 for band presence, 0 for band absence). CV: Ciliegino; SMR: San Michele Rosso; MO: Monachella; MA: Mascherino; PI: Pinto; TR: Tuvagliedda Rossa; SA: Suocera e Nuora.

	LOL2								LOL7							LOL8							LOL9							
	Bands								Bands							Bands							Bands							
Sample	1	2	3	4	5	6	7	8	1	2	3	4	5	6	7	1	2	3	4	5	6	7	1	2	3	4	5	6	7	8
**CV 1**	1	1	1	1	0	1	0	0	1	0	1	0	0	1	1	1	1	1	1	1	1	1	1	1	1	1	1	1	1	0
**CV 2**	1	1	1	1	0	1	0	0	1	0	1	0	1	1	1	1	1	1	1	1	1	1	1	1	1	1	1	1	1	0
**CV 3**	1	1	1	1	0	1	0	0	1	0	1	0	1	1	1	1	1	1	1	1	1	1	1	1	1	1	1	1	1	0
**CV 4**	0	1	1	1	0	1	0	0	1	0	1	0	1	1	1	1	1	1	1	1	1	1	1	1	1	1	1	1	1	0
**CV 5**	1	1	1	1	0	1	0	0	1	0	1	0	1	1	1	1	1	1	1	1	1	1	1	1	1	1	1	1	1	0
**MA 1**	0	1	1	1	1	1	1	1	1	0	1	0	1	1	0	1	1	1	1	0	1	1	1	0	0	1	1	1	1	1
**MA 2**	0	1	1	1	1	1	1	1	1	0	1	0	1	1	0	1	1	1	1	0	1	1	1	0	0	1	1	1	1	1
**MA 3**	0	1	1	1	1	1	1	1	1	0	0	0	1	0	0	1	1	1	1	0	1	1	1	0	0	1	1	1	1	1
**MA 4**	0	1	1	1	1	1	1	1	1	0	1	0	1	1	0	1	1	1	1	0	1	1	1	0	0	1	1	1	1	1
**MA 5**	0	1	1	1	0	1	1	1	1	0	1	0	1	1	0	1	1	1	1	0	1	1	1	0	0	1	1	1	1	1
**PI 1**	0	1	1	1	1	1	1	1	1	0	1	0	1	1	0	1	1	1	1	0	1	1	1	0	0	1	1	1	1	0
**PI 2**	0	1	1	1	1	1	1	1	1	0	1	0	1	1	0	1	1	1	1	0	1	1	1	0	0	1	1	1	1	0
**PI 3**	0	1	1	1	1	1	1	1	1	0	1	0	1	1	0	1	1	1	1	0	1	1	1	0	0	1	1	1	1	0
**PI 4**	0	1	1	1	1	1	1	1	1	0	1	0	1	1	0	1	1	1	1	0	1	1	1	0	0	1	1	1	1	0
**PI 5**	0	1	1	1	1	1	1	1	1	0	1	0	1	1	0	1	1	1	1	0	1	1	1	0	0	1	1	1	1	0
**SMR 1**	0	1	1	1	1	1	1	0	1	0	1	0	1	1	0	1	1	1	1	0	1	1	1	0	0	1	1	1	1	1
**SMR 2**	0	1	1	1	1	1	1	0	1	0	1	0	1	1	0	1	1	1	1	0	1	1	1	0	0	1	1	1	1	1
**SMR 3**	0	1	1	1	1	1	1	0	1	0	1	0	1	1	0	1	1	1	1	0	1	1	1	0	0	1	1	1	1	1
**SMR 4**	0	1	1	0	1	1	1	0	1	0	1	0	1	1	0	1	1	1	1	0	1	1	1	0	0	1	1	1	1	1
**SMR 5**	0	1	0	0	1	1	1	0	1	0	1	0	1	1	0	1	1	1	1	0	1	1	1	0	0	1	1	1	1	1
**TR 1**	0	1	1	1	1	1	1	0	0	0	1	1	1	1	0	1	1	1	1	0	1	1	1	0	0	0	0	1	1	1
**TR 2**	0	1	1	1	1	1	1	0	0	0	1	1	1	1	0	1	1	1	1	0	1	1	1	0	0	0	0	1	1	1
**TR 3**	0	1	1	1	1	1	1	0	0	0	1	1	1	1	0	1	1	1	1	0	1	1	1	0	0	0	0	1	1	1
**TR 4**	0	1	1	0	1	1	1	0	0	0	1	1	1	1	0	1	1	1	1	0	1	1	1	0	0	0	0	1	1	1
**TR 5**	0	1	1	0	1	1	1	0	0	0	1	1	1	1	0	1	1	1	1	0	1	1	1	0	0	0	0	1	1	1
**SA 1**	1	1	1	1	1	1	1	0	1	1	1	1	1	1	0	1	1	1	1	0	1	1	1	1	0	0	0	1	1	1
**SA 2**	1	1	1	1	1	1	1	0	1	1	1	1	1	1	0	1	1	1	1	0	1	1	1	1	0	0	0	1	1	1
**SA 3**	1	1	1	1	1	1	1	0	1	1	1	1	1	1	0	1	1	1	1	0	1	1	1	1	0	0	0	1	1	1
**SA 4**	1	1	1	1	1	1	1	0	1	1	1	1	1	1	0	1	1	1	1	0	1	1	1	1	0	0	0	1	1	1
**SA 5**	1	1	1	1	1	1	1	0	1	1	1	1	1	1	0	1	1	1	1	0	1	1	1	1	0	0	1	1	1	1
**MO 1**	1	1	1	1	1	1	1	1	1	1	1	1	1	1	0	1	1	1	1	0	1	1	1	1	0	0	0	1	1	1
**MO 2**	1	1	1	1	1	1	1	1	1	1	1	1	1	1	0	1	1	1	1	0	1	1	1	1	0	0	0	1	1	1
**MO 3**	1	1	1	1	1	1	1	1	1	1	1	1	1	1	0	1	1	1	1	0	1	1	1	1	0	0	0	1	1	1
**MO 4**	1	1	1	1	1	1	1	1	1	1	1	1	0	0	0	1	1	1	1	0	1	1	1	1	0	0	0	1	1	1
**MO 5**	1	1	1	1	1	1	1	1	1	1	1	1	1	1	0	1	1	1	1	0	1	1	1	1	0	0	0	1	1	1

	LOL10						LOL12								PHV06				PHV07			
	Bands						Bands								Bands				Bands			
Sample	1	2	3	4	5	6	1	2	3	4	5	6	7	8	1	2	3	4	1	2	3	4
**CV 1**	1	1	0	0	0	1	1	1	1	0	0	0	0	1	1	1	1	1	1	1	1	1
**CV 2**	1	1	0	0	0	1	1	1	1	0	0	0	0	1	1	1	1	0	1	1	1	1
**CV 3**	1	1	0	0	0	1	1	1	1	0	0	0	0	1	1	1	1	1	1	1	1	1
**CV 4**	1	1	0	0	0	1	1	1	1	0	0	0	0	1	1	1	1	1	0	0	0	0
**CV 5**	1	1	0	0	0	1	1	1	1	0	0	0	0	1	1	1	1	1	1	1	1	1
**MA 1**	1	1	0	0	0	1	1	1	1	0	0	0	0	1	1	1	1	1	0	1	1	1
**MA 2**	1	1	0	0	0	1	1	1	1	0	0	0	0	1	1	1	1	1	0	1	1	1
**MA 3**	1	1	0	0	0	1	1	1	1	0	0	0	0	1	1	1	1	1	0	1	1	1
**MA 4**	1	1	0	0	0	1	1	1	1	0	0	0	0	1	1	1	1	1	0	1	1	1
**MA 5**	1	1	0	0	0	1	1	1	1	0	0	0	0	1	1	1	0	1	0	1	1	1
**PI 1**	0	0	0	1	1	1	1	1	1	0	0	0	0	1	1	1	1	1	0	1	1	1
**PI 2**	0	0	1	1	1	1	1	1	1	0	0	0	0	1	1	1	1	1	0	1	1	1
**PI 3**	0	0	0	1	1	1	1	1	1	0	0	0	0	1	1	1	1	1	0	1	1	1
**PI 4**	0	0	0	0	0	1	1	1	1	0	0	0	0	1	1	1	1	1	0	1	1	1
**PI 5**	0	0	1	1	1	1	1	1	1	0	0	0	0	1	1	1	1	1	0	1	1	1
**SMR 1**	0	0	1	1	1	1	1	1	1	0	0	0	0	1	1	1	1	1	0	0	1	1
**SMR 2**	0	0	1	1	1	1	1	1	1	0	0	0	0	1	1	1	1	1	0	0	1	1
**SMR 3**	0	0	1	1	1	1	1	1	1	0	0	0	0	1	1	1	1	1	0	1	1	1
**SMR 4**	0	0	1	1	1	1	1	1	1	0	0	0	0	1	0	0	0	0	0	0	1	1
**SMR 5**	0	0	1	1	1	1	1	1	1	0	0	0	0	1	1	1	1	1	0	0	1	1
**TR 1**	0	1	0	0	0	1	0	0	0	0	0	1	1	0	1	1	1	1	0	1	1	1
**TR 2**	0	1	0	0	0	1	0	0	0	0	0	1	1	0	1	1	1	1	0	1	1	1
**TR 3**	0	1	0	0	0	1	0	0	0	1	1	1	1	0	1	1	1	1	0	1	1	1
**TR 4**	0	1	0	0	0	1	0	0	0	1	1	1	1	0	1	1	1	1	0	1	1	1
**TR 5**	0	1	0	0	0	1	0	0	0	0	0	0	0	0	1	1	1	1	0	1	1	1
**SA 1**	0	0	0	0	0	1	0	0	0	1	1	1	1	0	1	1	1	1	0	1	1	1
**SA 2**	0	1	0	0	0	1	0	0	0	1	1	1	1	0	1	1	1	1	0	1	1	1
**SA 3**	0	1	0	0	0	1	0	0	0	1	1	1	1	0	1	1	1	1	0	1	1	1
**SA 4**	0	1	0	0	0	1	0	0	0	0	0	0	0	0	1	1	1	1	1	1	1	1
**SA 5**	0	1	0	0	0	1	0	0	0	1	1	1	1	0	1	1	1	1	1	1	1	1
**MO 1**	0	1	0	0	0	1	0	0	0	0	0	1	1	0	1	1	1	1	1	1	1	1
**MO 2**	0	1	0	0	0	1	0	0	0	0	0	1	1	0	1	1	1	1	1	1	1	1
**MO 3**	0	1	0	0	0	1	0	0	0	0	0	1	1	0	1	1	1	1	1	1	1	1
**MO 4**	0	1	0	0	0	1	0	0	0	0	0	1	1	0	1	1	1	1	1	1	1	1
**MO 5**	0	1	0	0	0	1	0	0	0	1	0	1	1	0	1	1	1	1	1	1	1	1

Table S3. Primers used for ISSR analysis and their respective markers performance indexes. The ISSR primer name, 5’-3’ sequence (reported in Marotti et al. [2] and Pena-Ortega et al. [28]), and annealing temperature are listed together with their performance indexes: Number of Total Bands (NTB), Number of Monomorphic Bands (NMB), Number of Polymorphic Bands (NPB), Percentage of Polymorphic Bands (%P), Resolving Power (RP) and Polymorphism Information Content (PIC).

Primer name	Nucleotide sequence 5' -> 3'	Annealing temperature	NTB	NMB	NPB	%P	RP	PIC
LOL2	$(CT)_8GC$	37 °C	8	4	4	50.00	2.57	0.22
LOL7	$(GAGA)_3CC$	34 °C	7	3	4	57.14	2.29	0.24
LOL8	$(GT)_6CC$	34 °C	7	6	1	14.29	0.29	0.03
LOL9	$(CAC)_3GC$	34 °C	8	3	5	62.50	3.09	0.26
LOL10	$(GAG)_3GC$	34 °C	6	1	5	83.33	2.63	0.32
LOL12	$(GTG)_3GC$	34 °C	8	0	8	100.00	5.60	0.44
PHV06	$CCA(CT)_8$	37 °C	4	4	0	0.00	0.34	0.08
PHV07	$GTG(GT)_8$	38 °C	4	2	2	50.00	1.03	0.20

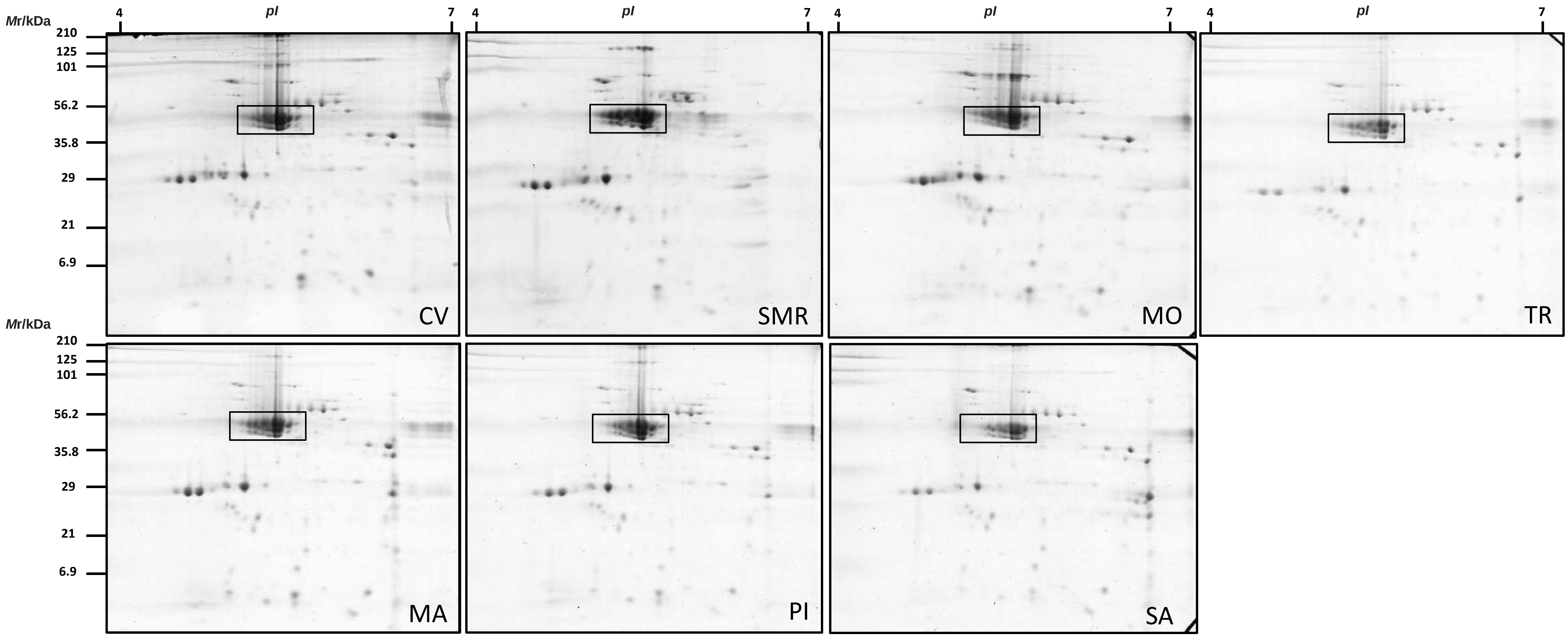

Figure S1. 2-DE profiles of common bean landraces. 2-DE was performed using 50 μg of total protein loaded on 7-cm long IPG strips, pH=4–7, and 15% (by mass) polyacrylamide gels. Gel regions containing phaseolin spots are enclosed in rectangles. CV: Ciliegino; SMR: San Michele Rosso; MO: Monachella; MA: Mascherino; PI: Pinto; TR: Tuvagliedda Rossa; SA: Suocera e Nuora.

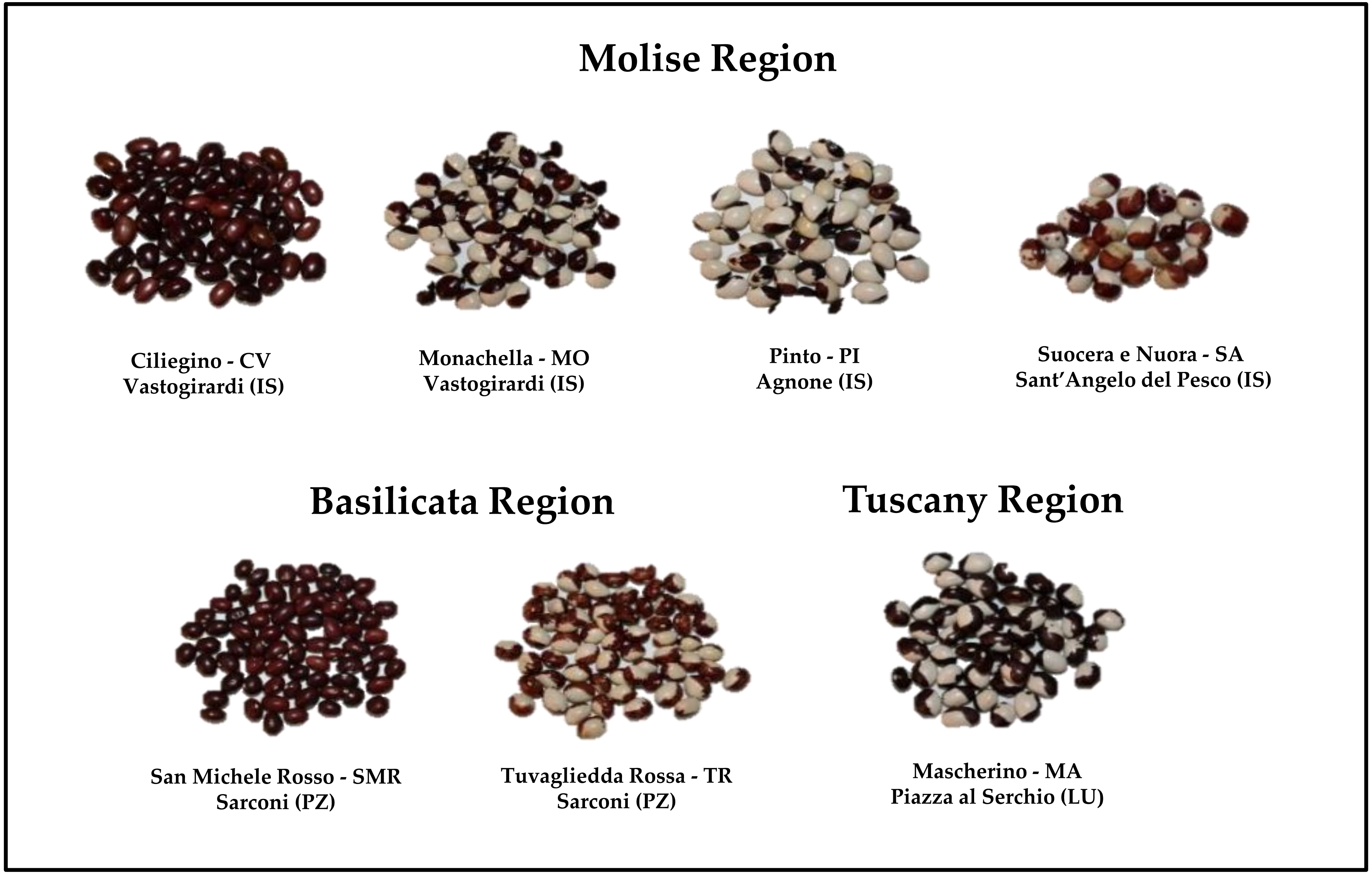

Figure S2. Autochthonous Italian Common bean seed accessions. Seed of the seven *Phaseolus vulgaris* L. landraces analyzed in the present study and cultivated in the Molise region (Italy) and in other two Italian Apennine regions (Tuscany and Basilicata – Italy).
